# Traffic jam within lymphocytes: A clinician’s perspective

**DOI:** 10.3389/fimmu.2022.1034317

**Published:** 2023-01-16

**Authors:** Smitha Hosahalli Vasanna, Jignesh Dalal

**Affiliations:** ^1^ Department of Pediatrics, Division of Pediatric Hematology Oncology, University Hospitals Rainbow Babies & Children's Hospital, Cleveland, OH, United States; ^2^ School of Medicine, Case Western Reserve University, Cleveland, OH, United States

**Keywords:** cellular trafficking, HLH Hemophagocytic lymphohistiocytosis, immune dysregulation, autophagy, hematopoietic stem cell transplantation

## Abstract

With the discovery of novel diseases and pathways, as well as a new outlook on certain existing diseases, cellular trafficking disorders attract a great deal of interest and focus. Understanding the function of genes and their products in protein and lipid synthesis, cargo sorting, packaging, and delivery has allowed us to appreciate the intricate pathophysiology of these biological processes at the molecular level and the multi-system disease manifestations of these disorders. This article focuses primarily on lymphocyte intracellular trafficking diseases from a clinician’s perspective. Familial hemophagocytic lymphohistiocytosis is the prototypical disease of abnormal vesicular transport in the lymphocytes. In this review, we highlight other mechanisms involved in cellular trafficking, including membrane contact sites, autophagy, and abnormalities of cytoskeletal structures affecting the immune cell function, based on a newer classification system, along with management aspects of these conditions.

## 1 Introduction

Intracellular trafficking is a highly sophisticated system that ensures the precise distribution and compartmentalization of cellular metabolites such as proteins and macromolecules. Metabolites are either internalized by means of endosomes (vesicular transport) or exported by means of exosomes *via* the secretory pathway. Numerous organelles, including the endoplasmic reticulum, Golgi apparatus, lysosomes, phagosomes, interorganelle interaction sites, and cytoskeleton, comprise the trafficking machinery. Nearly one-third of human genome-encoded proteins are exported from the endoplasmic reticulum (ER) to the Golgi apparatus, where they are sorted and transported to their destination. Defects in trafficking can affect cellular function and metabolism at various levels and can have adverse health effects. Any organ system in the body could be affected by membrane trafficking, but the nervous system is especially vulnerable due to its long axonal projections and necessity for precise spatiotemporal control over vesicular transport. Neurological disorders include several neurodevelopmental abnormalities, progressive encephalopathies with or without microcephaly, and neurodegenerative conditions in both children and adults. Parkinson’s disease, frontotemporal dementia, hereditary spastic paraparesis, amyotrophic lateral sclerosis, Charcot-Marie-Tooth disease, and genetic alterations affecting COP, TRAPP, VPS, RAB, and TANGO2 are a few examples (1–7). Nearly 346 genetic variants associated with cellular trafficking have been identified so far, with the number continuing to expand (8, 9).

Understanding the pathogenesis of cellular trafficking disorders requires a fundamental understanding of protein transport within cells. Through vesicle-mediated and non-vesicle-mediated trafficking, intracellular proteins are guided precisely to their target sites.

Vesicle-mediated trafficking involves proteins synthesized in the rough endoplasmic reticulum (RER) that are folded and transported to the Golgi apparatus. After undergoing post-translational modifications in the Golgi apparatus, these vesicles can be included as plasma membrane proteins (constitutive transport), expelled from the cell *via* exocytosis (regulated transport), or incorporated into lysosomes (endosomal transport). The coat proteins COPI, COPII, and Clathrin, are responsible for the transportation of vesicles inside the cell, especially to and from the Golgi apparatus. COP I coats vesicles traveling from the cis-Golgi to the RER (retrograde transport).COP II coats vesicles traveling from the RER to the cis-Golgi (anterograde transport), and Clathrin coats vesicles traveling from the Golgi to the lysosomes and from the plasma membrane to the lysosomes (10–14).

**Figure 1:**
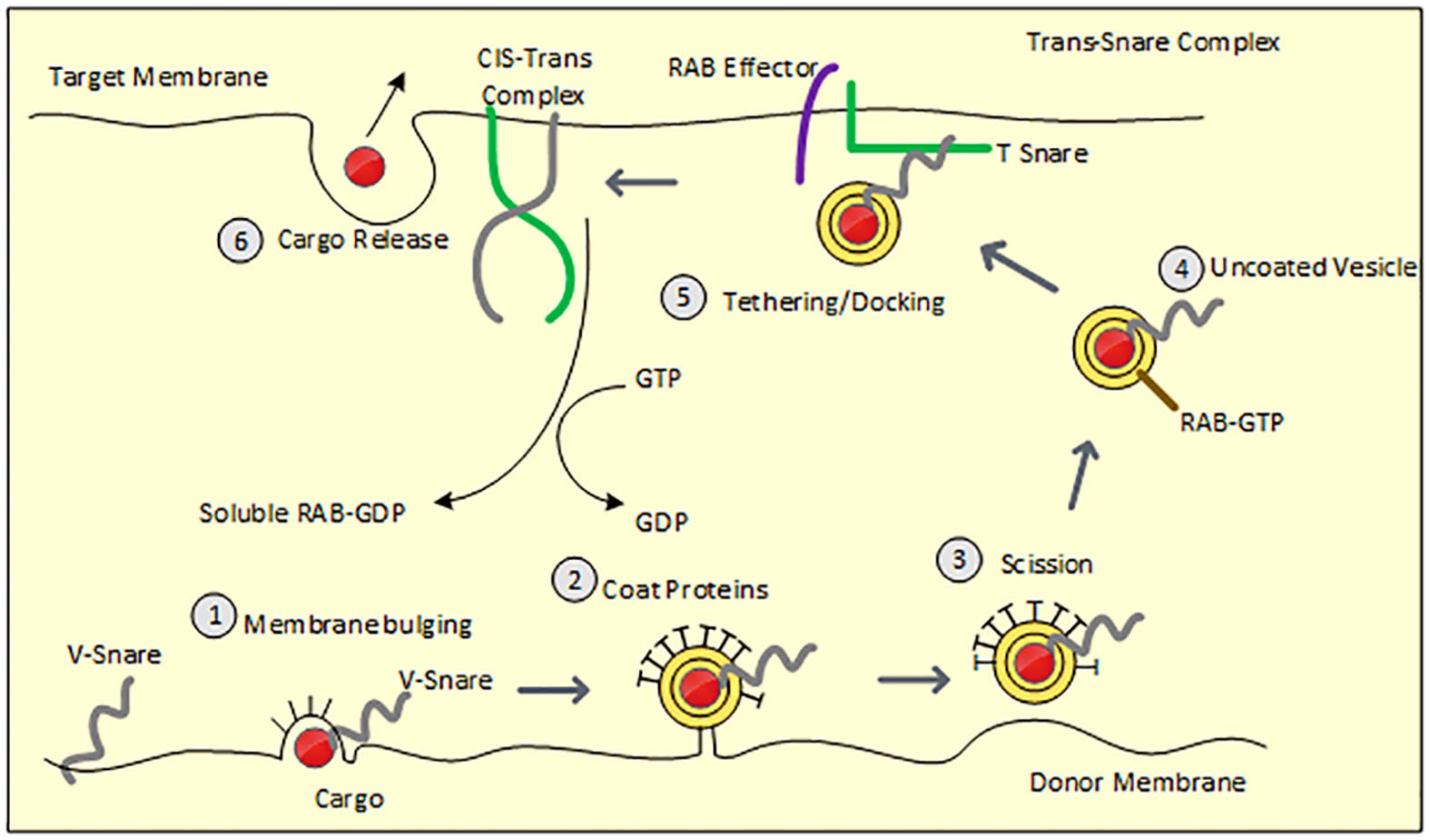
Demonstrates steps involved in vesicle budding and fusion. Figure 1 Membrane bulging: Recruited coat proteins line the donor membrane which bulges along with the assembly of cargo and V-SNARE protein. 2. Vesicle budding: Bulged membrane acquires circular appearance with concentrated cargo in the center and is only attached to the donor with a narrow neck. 3. Scission: vesicular neck is severed from the donor membrane 4. Uncoating of vesicles: Vesicle loses coat proteins within the cytosol and naked vesicle reaches the acceptor membrane *via* cytoskeletal movement 5. Tethering/Docking: V-SNARE forms an assembly with T-SNARE (acceptor membrane) along with the GTP-bound RAB effector to form a trans-SNARE complex resulting in fusion of the vesicular membrane with the acceptor membrane. Dissociated V-SNARE and T-SNARE from the vesicle form the Cis-Trans complex. 6. Cargo is released.

The cargo protein is transported *via* vesicular intermediates that originate from a donor compartment and merge with a recipient compartment. Cargo selection and vesicle budding are mediated by coat proteins, whereas vesicle targeting and fusion require SNARE proteins and Rab GTPases. **Figure 1** depicts the process of vesicle budding, docking, fusion, and cargo release.

Cellular trafficking diseases have been traditionally exclusively identified as membrane trafficking disorders in the context of vesicular transport. However, an Italian group of researchers has advised restructuring and categorizing these diseases based on recent knowledge of newer mechanisms involved in cellular trafficking (8).

- Membrane/vesicular trafficking within organelles
- Organelle – interorganelle trafficking or Membrane contact sites trafficking
- Autophagy
- Cytoskeleton-related
- Miscellaneous/others.

**Figure 2:**
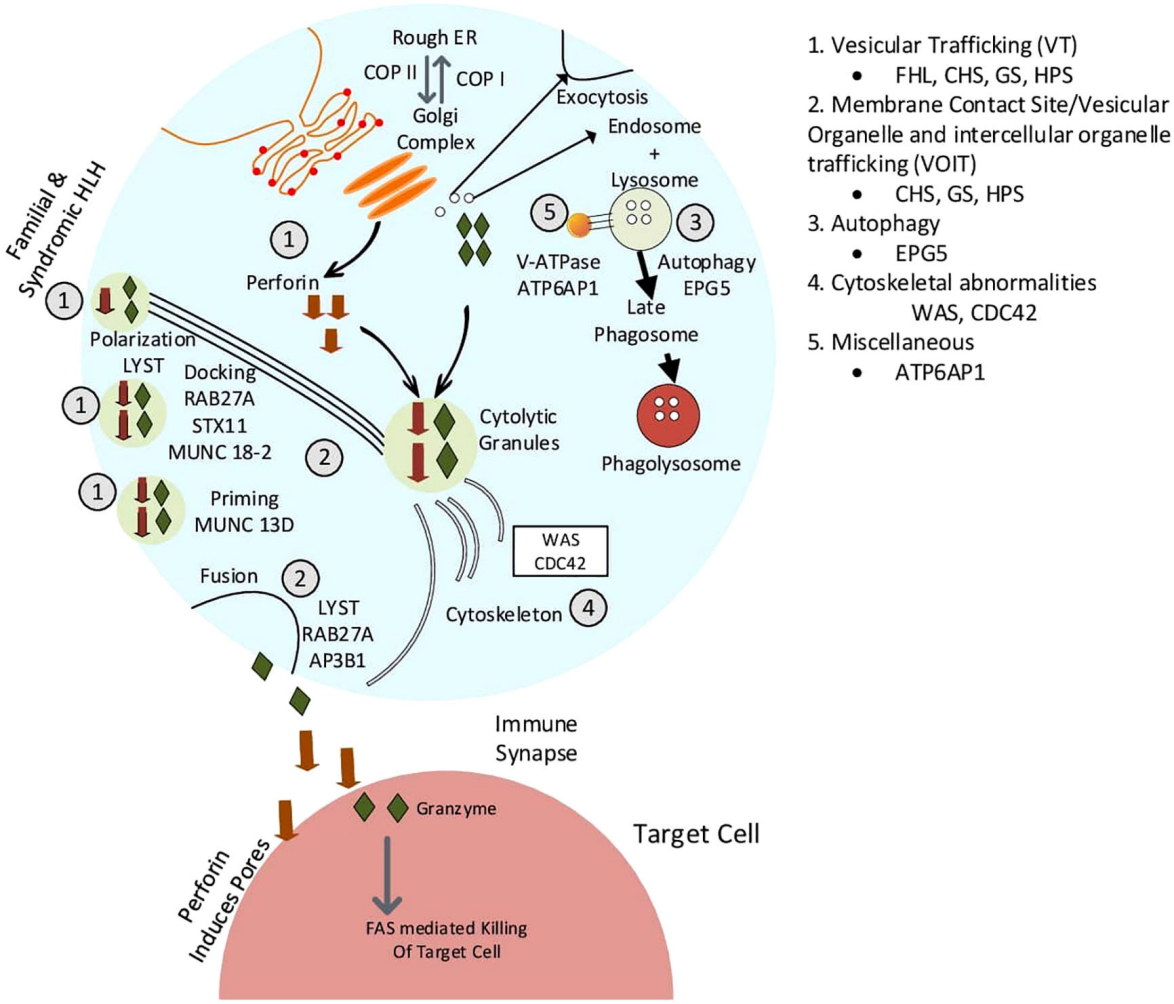
Pictorial representation of defects in membrane trafficking and associated diseases.

This article provides an overview of this new classification system, with with an emphasis on intracellular transport abnormalities emphasis on intracellular transport abnormalities in lymphocytes, particularly T lymphocytes and natural killer (NK) cells. Immune cells release cytokines, chemokines, and secretory lysosomal enzymes *via* the secretory pathway, mediating target cell death and inflammation. **Table 1** displays the classification of membrane trafficking disorders in lymphocytes based on the suggested mechanism by Garcia-Cazoria et al. and the related disorders (8). **Figure 2** is a pictorial representation of the pathways and related disorders.

**Table 1:** Pathophysiologic classification of cellular trafficking disorders in lymphocytes.

Pathophysiology of cellular trafficking	Diseases	Associated genes
1. Vesicular trafficking	Familial HLH (FHL)	PRF1, UNC3D, STX11, STXBP2, NBAS
	Syndromic HLH –Chediak Higashi Syndrome –Griscelli syndrome –Hermansky Pudlak syndrome	LYST, BLOC1S subtypes RAB27A AP3B1
	Lymphoproliferative disorders (XMEN)	MAGT1
	COPA syndrome/STING associated vasculopathy	TMEM17
	Other genetic diseases	DKC1, FLI1, HYOU, JAGN1, PACS1, TIC37, VIPAS39, VPS13B
2. Membrane Contact Site disorders	Griscelli Syndrome	RAB27A
	Hermansky Pudlak Syndrome	AP3BP1, AP3D1
	Chediak Higashi Syndrome	LYST
3.Autophagy	Vici syndrome	EPG5
4. Cytoskeletal abnormalities	Wiskott Aldrich Syndrome	WAS
	Takenouchi-Kosaki syndrome	CDC42
5. Others	Immunodeficiency type 47	ATP6AP1
	Autoimmune conditions	Systemic lupus erythematosus, Pustular psoriasis

## 2 Clinical manifestations and overview of trafficking disorders

### 2.1 Disorders of vesicular trafficking

Cytotoxic T lymphocytes (CTL) and NK cells play a crucial role in the elimination of virally infected and malignant cells. While the mechanisms of initial activation are distinct for each of these cell types, the ultimate phase of cytotoxicity is attributed to secretory lysosomal granules that release perforin and granzyme into the immunological synapse (IS) between the target and effector cells *via* exocytosis. Exocytosis involves the fusion of the cytolytic granule membrane with the plasma membrane, an action triggered by a protein complex called the soluble NSF attachment receptor (SNARE). Once it has been released into the synaptic space, perforin creates pores on the target membrane. These pores allow granzyme to enter the cell and activate the FAS pathway, which ultimately results in the apoptosis of the target cell.

HLH and the recently identified autoinflammatory/autoimmune disorder COPA syndrome alter the vesicular trafficking in lymphocytes, as discussed in the following section.

#### 2.1.1 Familial and syndromic HLH

HLH is a life threatening condition characterized by hyperimmune activation due to aberrant lymphocyte cytotoxicity in CTL and NK cells, resulting in a failure to eliminate the effector cell. This sets up a vicious loop of constant stimulation of CTL and NK cells, which releases proinflammatory cytokines, resulting in a cytokine storm. Clinical manifestations include persistent fever, pancytopenia, splenomegaly, hypofibrinogenemia, coagulopathy, liver failure, central nervous system involvement, high inflammatory markers like ferritin, soluble CD25, and absent or low NK cell function.

Inherited or familial HLH (FHL) occurs due to an underlying genetic defect and includes HLH as the predominant manifestation, whereas the etiology of secondary HLH includes infections (most common), malignancies, and autoimmune or autoinflammatory conditions (15–17). Clinical syndrome strongly mimicking or overlapping HLH caused by rheumatological disorders, particularly systemic lupus erythematosus, is known as macrophage activation syndrome (MAS). FHL is a genetically heterogeneous disorder that results from mutations in genes involved in the secretory lysosome-dependent exocytosis pathway, such as abnormalities in vesicle maturation, docking, priming, and fusion. There are five subtypes of FHL (1-5) defined, although only subtypes 2-5 have documented genetic mutations. Perforin gene mutations are associated with FHL-2, and genetic variants in FHL 3-5 affect proteins involved in transport, membrane fusion, or exocytosis of perforin-containing lytic granules, such as UNC13D (FHL-3), STX-11 (FHL-4) and STX-BP2 (FHL-5). FHL 1 has been mapped to chromosome 9q21.3-22, although the specific gene has not yet been identified (18).

In addition to the HLH risk, syndromic forms of HLH such as Chediak Higashi Syndrome (CHS), Griscelli Syndrome (GS), and Hermansky Pudlak Syndrome (HPS) exhibit pigmentary abnormalities of the skin, hair, and oculocutaneous albinism, appearing as light skin and silver-colored hair (19–25). Additional clinical manifestations of CHS include progressive neurologic abnormalities, mild coagulation defects, pathognomonic giant cytoplasmic granules in leucocytes, and platelets on peripheral smear examination. GS manifestations include cytopenias and neurologic abnormalities secondary to cerebral lymphohistiocytic infiltration, while HSP2 manifestations include platelet dysfunction (26, 27).

Both X-linked lymphoproliferative disorders (XLP1 and XLP 2) and XMEN disease [X-linked recessive condition, magnesium defect, Epstein- Barr virus (EBV) infection, and neoplasia] are at high risk of HLH, particularly following EBV infection (28–30).

#### 2.1.2 COPA syndrome/STING associated vasculopathy

COPA syndrome is an autosomal dominant immune dysregulation disorder named after the mutated gene that was discovered in 2015. The COPA gene encodes for the COP-I (COP-alpha) subunit of the coatomer complex, which is responsible for the retrograde transportation of proteins from the Golgi to the ER. The mutation has little effect on the overall level of COP expression in the cell, but it disrupts protein transport, increases ER stress, and activates the unfolded protein response leading to autophagy, thereby triggering autoimmune and autoinflammatory responses (31, 32).

COPA syndrome is an interferonopathy characterized by arthritis, interstitial lung disease with pulmonary hemorrhage, and immune complex-mediated renal disease. Nearly 75% of the patients are under the age of five, and almost all of them have lung involvement, while 50% have arthritis and 25% have renal disease. On a computed tomography (CT) of the chest, ground glass opacities, cysts, and bronchial wall thickening can be observed; a restrictive pattern can be seen on pulmonary function tests, and histologically, lymphocytic interstitial pneumonia and follicular bronchiolitis are common associations. Lepelley et al. demonstrated the presence of lung infiltrating CD5+ T lymphocytes and follicular CD20+ B cells within the lymphoid infiltration by immunohistochemical stains (33). In patients with COPA syndrome, increased Th17 cells and Th17 stimulating cytokines such as IL-1β, IL-6, and IL-23 along with reduced CD4 lymphocytes have been reported. Due to the widespread expression of COPA, Vece et al. hypothesized that abnormal COPI function occurs in both immune and somatic cells (31). Lung autoimmunity is uncommon outside of SLE and ANCA-associated vasculitis in children, and suspicion should be raised for this specific immune dysregulation syndrome. Three different presentations of COPA syndrome described in literature include pulmonary hemorrhage in a young patient, interstitial lung disease in a teenager and vasculitis. The latter two usually have a family history of other autoimmune conditions like rheumatoid arthritis or familial vasculitis.

Although the exact pathophysiology of COPA syndrome is unknown, available studies indicate that a malfunction in COPI transport is responsible for ligand-independent activation of STING and that COPA regulates STING transport at the Golgi to maintain immune homeostasis (34). The cGAS-STING signaling pathway plays an important role in inflammation and innate immunity by sensing and regulating cellular responses to microbial and host-tissue-derived DNA through the release of proinflammatory cytokines, type 1 interferons, and other antiviral proteins. STING then activates the downstream protein kinase TBK1, which phosphorylates and activates interferon regulatory factor 3 (IRF3), the essential transcription factor that drives type I interferon production.

### 2.2 Disorders associated with oragnelle/interorganelle trafficking defects/membrane contact sites

Inter-organelle membrane contact sites (MCS) are conventionally defined as areas of proximity between the two organellar membranes that are established by specific proteins called tethers. Contact between organelles with identical membranes is referred to as “homotypic,” whereas contact between organelles with distinct membranes is referred to as “heterotypic. Some examples of heterotypic contact include ER-plasma membrane (PM), ER-mitochondria, ER-lysosomes, and ER-Golgi. Recent research has discovered an unparalleled depth and breadth of information regarding MCS.

Syndromic HLH conditions listed below have defects in vesicular trafficking and organelle/interorganelle trafficking defects, also called membrane contact site disorders, either alone or in combination.

#### 2.2.1 Chediak Higashi syndrome

CHS is caused by mutations in the LYST (Lysosomal Trafficking Regulator) gene, which encodes a highly conserved LYST protein with four domains: an amino-terminal ARM/HEAT domain, a PH (Pleckstrin-homology) domain, a BEACH (Beige and Chediak-Higashi) domain, and seven carboxy-terminal domains (WD-40). Physiologically, endosomes fuse with lysosomes and split apart after delivering their contents, thereby facilitating the maturation of lysosomes while maintaining their size. Various beige mouse models have led to the hypothesis that LYST either promotes lysosome fission or heterotypic membrane fusions (late endosomes with mature lysosomes) (35, 36). Few studies, including one by Holland et al., found that LYST mutations did not interfere with normal lysosomal fusion and resulted in normal autophagosome formation or endosomal degradation (37, 38). Due to the large size of the granules, lysosomal exocytosis, and fusion with the plasma membrane were reported to be abnormal in CHS/beige fibroblasts (39, 40). Melanocytes, CTL/NK cells, and platelet dense granular maturation are altered in CHS, causing partial oculocutaneous albinism, recurrent infections, an accelerated phase of HLH, and bleeding manifestations. High LYST expression in the brain explains the progressive neurological symptoms observed in CHS.

#### 2.2.2 Griscelli syndrome

GS is an AR disorder caused by mutations in the MYO5A, MLPH, and RAB27A genes, which are respectively responsible for GS types 1, 2, and 3. Type 2 GS exhibits immunodeficiency (defect in granule exocytosis by CTL) and silver hair (abnormal melanosome transport) with the HLH phenotype, whereas Type 1 predominantly has neurologic manifestations. Rabs are Ras-like small GTPases, and they function as molecular switches that can toggle between an active GTP-bound state and a resting GDP-bound state. This allows them to control multiple steps in the secretory process, such as priming, tethering, docking, and fusion.

#### 2.2.3 Hermansky Pudlak syndrome

HPS type 2 is due to a mutation in the adapter protein 3 subunit (AP-3) of the membrane complex that manifests with immune dysregulation, whereas bleeding disorders are the predominant manifestation of other types of HPS. AP-3 is a heterotetrameric complex that is required for the formation of vesicles on early endosomes. It recruits cargo proteins by sorting cytoplasmic signals, packages them into clathrin-coated vesicles, and transports them to late endosomal compartments (41, 42). AP-3 mutation results in protein missorting and misrouting, leading to pigmentation abnormalities, neutropenia, and increased risk of HLH.

### 2.3 Autophagy

Autophagy, or “self-eating,” is a crucial intracellular degradation system that transports cytoplasmic materials, including damaged organelles, undesired proteins, and intracellular pathogens, to the lysosomes and degrades them within. Microautophagy, macroautophagy, and chaperone-mediated autophagy are the three types of autophagy. Of these, macroautophagy is the most conserved and is essential for numerous physiological functions such as the elimination of damaged proteins and organelles, defense against infections, cellular development and differentiation, metabolic demand adaptability, and tumor growth progression (43, 44). Macroautophagy is a sequential process that begins with a double isolation membrane or phagophore in the cytosol and progresses to a spherical autophagosome that contains undesired cellular components. The autophagosome then fuses with the outer membrane of the lysosome to form the autolysosome or phagolysosome, and the internal material is degraded and recycled.

Since the discovery of the autophagy-related gene (ATG) in 1990 and its role in cellular homeostasis, numerous other genetic mutations associated with impaired autophagy have been discovered, including BECN1 (breast and ovarian cancer), CLEC16A (diabetes and multiple sclerosis), CTNS (Cystinosis), EPG5 (Vici syndrome), and GBA1 (Gaucher’s disease). Vici syndrome, which is an autophagy disorder with multi-system involvement and abnormal lymphocyte trafficking,is discussed below.

#### 2.3.1 Vici syndrome

Vici Syndrome (OMIM242840) is a rare, autosomal recessive disorder that occurs due to a mutation in EPG5, a crucial autophagy regulator located on chromosome 18q12.3 (45). The disorder was first described in 2013 and is a typical example of congenital disorders of autophagy (46, 47). Although a multi-system disorder, the five defining features include callosal agenesis, cataracts, cardiomyopathy, hypopigmentation, and combined immunodeficiency. Most individuals exhibit severe developmental delay and acquired microcephaly; however, a subset can demonstrate neurodegeneration and regressive early infancy-acquired milestones. In addition to corpus callosal agenesis, other abnormalities on brain imaging include cortical and cerebellar malformations and aberrant thalamic signals on MRI that are similar to those seen in other lysosomal storage disorders. Most patients have pale skin and blond hair relative to their ethnic origin. In older children, coarsening of facial features, thick lips, and macroglossia have been reported. Vici syndrome is a progressive condition with a median age of survival of 42 months. So far, 40 different private mutations in the EPG5 gene in Vici syndrome have been reported (48).

Cullup et al. evaluated the role of autophagy in Vici syndrome, hypothesizing elevated p62/SQSTM1 and Nbr1 in atrophic muscles indicated a block in autophagy. Patient-derived and healthy muscle fibroblasts were exposed to rapamycin (an autophagy inducer) and bafilomycin (an autophagy inhibitor) and demonstrated that early autophagy steps were unaffected; however, they demonstrated a severe deficit in autophagosomal clearance in Vici syndrome. Autophagy-inhibiting drugs have been tested in clinical trials (49).

### 2.4 Cytoskeletal abnormalities

The cargo-containing vesicle is directed to the target membrane *via* cytoskeletal structures like microfilaments and microtubules (heterodimers of α and β tubulins) and motor proteins like actin, myosin, dynein, and kinesin. The coordinated action of the actin and microtubule cytoskeletons, as well as intracellular vesicle trafficking, are critical in normal T cell function, such as membrane receptor localization, triggering, and intracellular signal modulation. Actin cables serve as routes that transport secretory vesicles to the cell’s apical terminals.

#### 2.4.1 Wiskott Aldrich syndrome

Wiskott-Aldrich protein is a type-1 actin nucleation promotion factor that regulates the Arp 2/3 complex (50). Members of the WAS protein family include WASP, N-WASP, WAVE, and WASH. WASH is believed to play a major role in early and late endosomal sorting and autophagy maturation by regulating the association between vesicles and actin (51, 52). Additionally, WASP promotes the attachment of secretory vesicles to the plasma membrane and is responsible for transmitting signals from the cell surface to the actin skeleton (53).

WAS is a rare x-linked recessive disorder characterized by microthrombocytopenia, eczema, and recurrent infections due to immunodeficiency, autoimmunity, and lymphoid malignancies (54, 55). WASP deficiency results in cytoskeletal defects that compromise multiple aspects of normal cellular activity, including proliferation, immune synapse formation, phagocytosis, cell adhesion, and migration (54). There is a strong genotype/phenotype correlation in WAS, with WASP- (complete absence) resulting in a classic WAS, and WASP+ (residual protein expression) resulting in the minor clinical phenotype of X-linked thrombocytopenia (XLT) with only minor eczema and thrombocytopenia. The classical triad includes thrombocytopenia, eczema, and recurrent infections. A clinical scoring system (score 1–5) helps to define severity; a score of 1-2 indicates the XLT phenotype, 3–4 indicates the classical WAS phenotype, and a score of 5 indicates autoimmunity or malignancy (56).

#### 2.4.2 CDC42 mutation

The CDC42 (Cell Division Cycle 42) gene encodes the Cdc42 protein, which belongs to the Rho GTPase family. Cdc42 interacts with multiple target proteins, including WASP, p21-activated kinases (PAKs), and FMNL2 (a formin-like protein), which are implicated in actin cytoskeletal organization as well as vesicle trafficking, motility and migration, and the maintenance of cell polarity (57). Affected CDC42 results in a distinct clinical phenotype with neurodevelopmental, hematologic, and immunologic abnormalities with increased predisposition to infections and malignancies.

Takenouchi-Kosaki syndrome (OMIM #616737) is a recently described syndrome (in 2015) with autosomal dominant inheritance. The cardinal features include intellectual disability and macrothrombocytopenia. Other manifestations include camptodactyly, structural brain abnormalities, congenital heart disease, sensorineural hearing loss, hypothyroidism, lymphedema, and recurrent infections (58). It is caused by a mutation in CDC42, which is essential for the regulation of the cell cycle, endocytosis, and the formation of the actin cytoskeleton. Electron microscopic examination of platelets in patients with Takenouchi-Kosaki syndrome revealed giant platelets with an increase in vacuoles. However, overt bleeding has not been reported despite the giant platelets.

Asiri et al. described pancytopenia, recurrent infection, poor wound healing, and heterotopia of the brain due to a novel CDC42 mutation (59). Further, a unique hematological/autoinflammatory syndrome (NOCARH syndrome) was reported by Lam et al. in four patients with dyshematopoiesis and HLH due to the CDC42 mutation (60). There is also a case report of an 11-year-old patient with syndromic immunodeficiency who developed HLH with a subsequent diagnosis of Hodgkin lymphoma and harbored p.Cys81Tyr, CDC42 mutation (61). Additionally, four patients with a novel CDC42 mutation and an autoinflammatory picture with a MAS-like phenotype have been reported. Three of these four patients had heterozygous LYST, UNC13D, and WAS mutations (62).

### 2.5 Miscellaneous

These conditions have multiple mechanisms of abnormal trafficking.

#### 2.5.1 Immunodeficiency type 47

Immunodeficiency type 47, also known as congenital disorder of glycosylation type II, is an inborn error of immunity (IEI) due to a mutation in the ATP6AP1 gene. The ATP6AP1 gene encodes Ac45, an accessory protein of the H+ ATPase or V (vacuolar) ATPase multi-domain protein complex. The V-ATPase mediates the acidification of membrane-bound intracellular organelles, mainly the lysosomes and Golgi. The cytosolic V1 domain hydrolyzes ATP, while the V0 transmembrane domain pumps protons through a rotatory mechanism into the lumen, causing luminal acidification (63). Consequently, it is suggested that the origin of this disease is a mutation in ATP6AP1 with defective Golgi acidification leading to Golgi-mediated glycosylation defects.

This X-linked recessive condition was originally described as hepatopathy, which would likely manifest as neonatal-onset jaundice and liver failure, and cognitive impairment. With the identification of new cases, additional manifestations, including immunodeficiency and hypogammaglobulinemia, hepatosplenomegaly, hearing loss, cutis laxa, and pancreatic insufficiency, have been reported as part of the spectrum.There have been only 18 cases of ATP6AP1-CDG-related disorders reported in the published literature. Barua et al. reported identical twins with the ATP6AP1 mutation who developed acute liver failure in the neonatal period and had prenatal onset cystic hygroma, an atrial septal defect, and ventriculomegaly, as well as postnatal onset connective tissue anomalies, pectus carinatum, and hypospadias (64). Gumm et al. have described two male siblings with rapidly progressive infantile onset cirrhosis, one of whom received a successful liver transplant (LT) at three years and ten months of age, while the other succumbed while awaiting LT (65). Neonatal-onset jaundice with an elevation of maternal alpha-fetoprotein levels without apparent cause should raise suspicion for this disorder and requires a genetic work-up.

## 3 Diagnosis

Due to the rarity of cellular trafficking disorders and the presence of multiple overlapping symptoms with other common conditions, a high index of suspicion is required to establish a correct diagnosis. Although genetic tests confirm a precise diagnosis, clinical presentation and rapid-turnaround laboratory tests may indicate a provisional diagnosis while awaiting genetic tests.

HLH can mimic sepsis/systemic inflammatory response syndrome. Diagnosis of HLH is established when a molecular diagnosis consistent with HLH is present, or five of the eight HLH-2004 criteria are met [fever ≥ 38.5°C, splenomegaly, cytopenias affecting at least 2 of the 3 lineages in peripheral blood (Hemoglobin < 9 g/dL in infants < 4 weeks: Hemoglobin < 10 g/dL, Platelets < 100 × 103/mL, Neutrophils < 1 × 103/mL), Hypertriglyceridemia (fasting, >265mg/dL) and/or hypofibrinogenemia (< 150 mg/dL), Hemophagocytosis in bone marrow, spleen, lymph nodes, or liver, Low or absent NK-cell activity, Ferritin > 500 ng/mL, Elevated sCD25 (α-chain of SIL-2 receptor) more than 2SD from the mean].

Like CTL and NK cells, melanocytes share the principle of lysosomal secretion. The absence of giant granules on the peripheral smear, the pattern of melanocyte clumping, and immunological evaluation can help differentiate CHS and GS. Hair microscopy in CHS shows evenly distributed melanin granules of regular diameter but bigger than those seen in normal hair. GS shows unevenly large melanin granules, mostly located in the vicinity of the medullary zone (66, 67).

HLH-2004 criteria have many pitfalls, including unknown sensitivity and specificity, especially for secondary HLH, as they were originally developed for diagnosing familial HLH. Reduced perforin, granzyme B, SLAM-associated proteins (SAP), and X-linked inhibitor of apoptosis protein (XIAP) expression by immunologic studies indicate genetic abnormalities in the perforin gene mutation, UNC13-D, SH2D1A, and XIAP, respectively whereas abnormal CD107a degranulation assays indicate STX11, STXBP2, and RAB27A mutations. Consequently, these tests could be utilized as a decision-making tool to direct targeted sequencing for precise genetic diagnosis. Whole exome sequencing (WES) or whole genome sequencing must be performed if clinical suspicion for HLH is high but targeted sequencing fails to identify the genetic abnormality. Rubin et al. compared the NK cell cytotoxicity assay, NK cell perforin, and CD107a expression in a large HLH cohort and discovered that perforin and CD107a expression were more sensitive and not less specific than NK cell cytotoxicity tests for screening genetic HLH, with reported sensitivities of 59.5%, 96.6%, and 93.8% and specificities of 72.0%, 99.5%, and 73%, respectively (68). Chinn et al. published their experience of genetic testing on HLH subjects prospectively over 17 years; 101 of the total 122 subjects underwent genetic testing. Targeted sequencing identified biallelic FHL gene defects in 19% of subjects, which correlated with infantile presentation (p < 0.0001). The majority of HLH in adults is secondary, as opposed to genetic in young children. Therefore, while temporary NK cell abnormalities are possible, cytotoxicity or degranulation abnormalities decrease with age. Carvelli et al. found an A91V variant monoallelic perforin mutation in over fifty percent (n= 68) of their adult cohort (69). Whole exome sequencing showed that 28 of 48 patients had abnormalities, such as inborn errors of metabolism (IEI) and immune dysregulation or lymphoproliferative disorders (Omenn syndrome, chronic granulomatous disease, and autoimmune lymphoproliferative syndrome), as well as new mutations in dysregulated inflammasome activity (monoallelic NLRC4 and NLRP12 and biallelic NLRC4, NLRP3, and NLRP13) (70). Amman et al. (the German group) prospectively used a flow cytometry-based immunological assay among 290 children with suspected HLH (68). They demonstrated accurate prediction of PRF-1, XLP1, and XLP2 in 29 patients by flowcytoemtry and among 85 patients with defective NK degranulation; 70 underwent Sanger sequencing with a resultant genetic diagnosis in 79% (55 patients). WES was performed in eight patients with negative results on targeted sequencing, revealing mutations in cytotoxicity genes in three patients and metabolic disease in one (71).

HLH is a hyperinflammatory phenotype rather than a single disease, wherein heterogeneous triggers, including infections, rheumatologic conditions, malignancies, and genetic HLH, culminate in a final common pathway of overactive T cells and macrophages that secrete enormous amounts of proinflammatory cytokines, such as interferon (IFN)-gamma, interleukin (IL)-1-beta, IL-2, IL-6, IL-10, IL-18, and tumor necrosis factor (TNF). The resultant cytokine storm is responsible for the clinical and pathologic manifestations of HLH. The umbrella phrase “cytokine storm syndrome” encompasses the wide range of aforementioned hyperinflammatory conditions. Multi-system inflammatory syndrome in children (MIS-C) secondary to coronavirus disease (COVID-19) also resulted in a similar clinical phenotype of hyperinflammation, especially in children (72). Genetic mutations TLR3, TLR7, and IRF7-dependent and neutralizing autoantibodies inhibited type I interferon signaling in COVID-19 patients who had severe disease (73). Separate sets of criteria have been established for MAS secondary to SLE and MAS secondary to juvenile idiopathic arthritis (74). Increased cytokines in MAS include macrophage- and monocyte-derived tumor necrosis factor (TNF) and interleukins such as IL-1, IL-6, and IL-18. In a febrile hospitalized child, hyperferritinemia and elevated serum ferritin to erythrocyte sedimentation rate are surrogate markers for the cytokine storm. HScore is a validated scoring system for reactive HLH developed by Fardet et al. It comprises a total of nine variables: underlying immunosuppression, fever, organomegaly, triglycerides, ferritin, aspartate aminotransferase, fibrinogen, cytopenia, and evidence of hemophagocytosis in bone marrow. In their cohort of both pediatric and adult patients, the median Hscore of reactive HLH patients was 230, and the predicted HLH probability from an HScore ≤90 was 1%, whereas an HScore ≥250 predicted an HLH probability of >99% (75).

**Figure 3:**
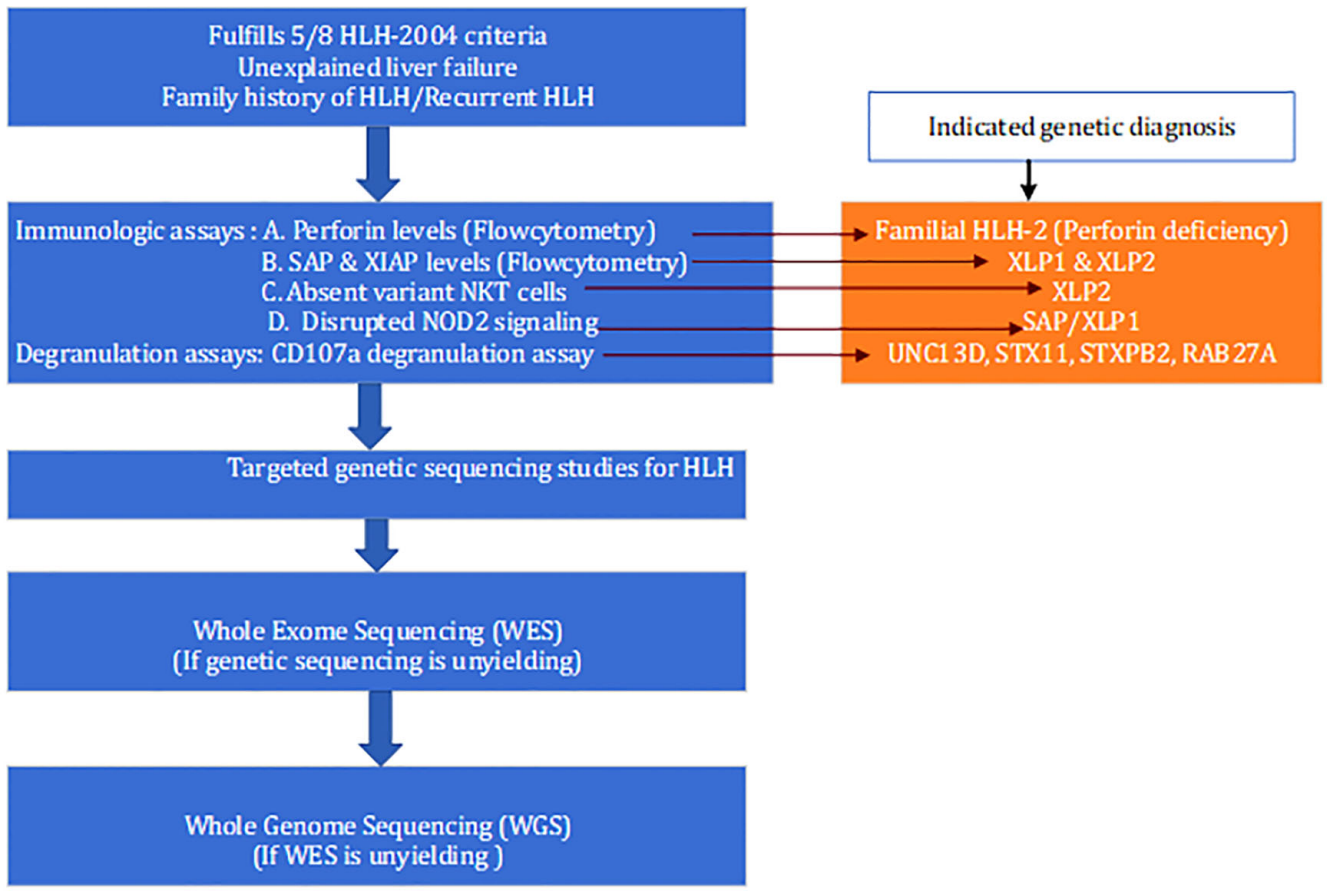
Proposed diagnostic algorithm for the diagnosis of suspected primary HLH.

Assessment of cytolytic function should be the second line of testing when clinical and laboratory evidence for HLH is convincing; followed by targeted genetic testing. However, it is plausible that targeted testing alone will not be sufficient to diagnose all cases of hereditary HLH. Hence, when these tests are negative, whole exome sequencing should be performed. Furthermore, genomic stratification of secondary HLH into molecularly defined phenotypes enables more targeted treatment. Even with WES, Chinn et al. were unable to diagnose HLH genetically in nearly half of the patients. In a cohort of infants with FHL, nearly half of the mutations were discovered in non-coding regions. The coding region or exome comprises only 2% of the entire genome; therefore, WGS is required in patients with a strong clinical suspicion of HLH in order to identify structural variants and non-coding region mutations. A proposed diagnostic algorithm for suspected primary HLH is depicted in **Figure 3**. A turnaround time of 6–8 weeks for the WGS is the most significant disadvantage that could delay definitive therapy.

COPA syndrome should be suspected in younger patients with autoimmune lung disease and diffuse alveolar hemorrhage, especially in the setting of a family history of rheumatological diseases. Autoimmune labs (ANA, c-ANCA, p-ANCA, RA factor), inflammatory markers like ESR and CRP, immunoglobulin assessment, immunophenotyping, and organ-specific markers like serum creatinine, proteinuria, liver enzymes, and thyroid function tests should be obtained for a baseline and assessment of therapy response. Specialized tests to assess the interferon pathway using the IFN alpha protein are available only as research tests. Depending on the clinical situation, COPA gene mutations can be found using targeted Sanger sequencing, WES, or WGS.

Absence of corpus callosum, cataracts, hypopigmentation, cardiomyopathy, immune dysfunction, profound developmental delay, progressive microcephaly, and failure to thrive have a 97% specificity and an 89% sensitivity for a positive EPG5 genetic test in Vici syndrome. Rare EPG5 variations of unclear importance may require functional autophagy studies in fibroblast cultures, which are only available for research purposes. Other diseases like WAS, Takenouchi-Kosaki, and immunodeficiency type 47 are diagnosed with genetic sequencing, WES, or WGS.

## 4 Treatment

Clinical manifestations of lymphocyte trafficking disorders range from severe cytokine storm in HLH to severe lung bleeding in COPA syndrome to life-threatening hemorrhage or infection in WAS and CDC42 mutation subtypes. As Vici syndrome and Takenouchi-Kosaki syndrome manifest as neurodevelopmental or neuroregressive disorders, a multimodal approach is required. This approach may include anticonvulsants, dietary support through a nasogastroic or gastric tube, physical therapy, occupational therapy, and speech therapy. Treatment for specific disorders is described in depth below.

HLH therapy aims to suppress life-threatening inflammation by suppressing the immune system and eliminating activated T-cells and macrophages. The decision to start therapy depends on the clinical stability, degree of inflammation (ferritin levels and stability of liver enzymes), and underlying trigger. Patients with primary HLH are predicted to survive only a few months if left untreated. Sick patients need stabilization and supportive care such as blood products, an intensive care stay, inotropic support, and respiratory support. With EBV being the most common trigger even in genetic HLH, viral infections may necessitate chemotherapy with rituximab in addition to standard chemotherapy. Based on HLH-94 and HLH-2004 Histiocyte Society clinical trials, dexamethasone, cyclosporine A, and etoposide for 8-week induction therapy, followed by HSCT as a consolidative therapy for familial/genetic, persistent, or recurrent HLH, have been the standard treatment approach (76). Etoposide, a topoisomerase II inhibitor, selectively eliminates pathologically activated T cells in HLH. A chemotherapy-sparing regimen with anti-thymocyte globulin (ATG) and prednisone was used for over 15 years in Paris with a > 70% remission rate. Though anakinra (recombinant human IL-1 receptor antagonist) had been mainly used by rheumatologists in the setting of macrophage activation syndrome (MAS), more recently, some centers have been using a combination of anakinra, intravenous immunoglobulin (IVIG), and steroids as a chemotherapy-sparing approach, especially in secondary HLH (77–80). IVIG inhibits complement activation, blocks antibody and macrophage Fc receptors, and neutralizes cytokines (81).In a retrospective study, the combination of IVIG and steroids was associated with a 75% resolution rate and an 82.5% 6-month OS in adults with secondary HLH, which was primarily used in the setting of overwhelming infections (82). Early administration of anakinra is postulated to stabilize the hyperinflammatory state in HLH, encouraging its use as a bridging therapy. The lack of comparative studies of anakinra vs. standard HLH therapy is the major limiting factor in assessing its effectiveness.

Chronic granulomatous disease (CGD) is an IEI characterized by a deficient respiratory burst mechanism, and interferon-gamma therapy is utilized to enhance it. Due to the incapacity of host phagocytes to eliminate infections, CGD patients are prone to developing an HLH-like phenotype, which, when paired with the persistent production of proinflammatory cytokines, could result in a cytokine storm. In such circumstances, interferon therapy could be counterintuitive and should be discontinued.

HSCT is the definitive treatment for genetic HLH and relapsed/refractory HLH in the absence of appropriate response to chemoimmunotherapy. A homozygous or compound heterozygous mutation must be demonstrated in genetic studies, proving the genetic diagnosis of HLH before HSCT. The role of HSCT in heterozygous patients is not clearly characterized and depends on recurrence or responsiveness to HLH therapy. The timing of the transplant in FHL is crucial, as delay increases the risk of irreversible CNS damage. A significant reduction in pre-HSCT mortality was reported between the HLH-2004 and HLH-94 studies (19% vs. 29%, respectively); however, there was no difference in overall survival (66% vs. 64%) (83). Both studies used busulfan containing myeloablative regimen for conditioning. Matched unrelated donor outcomes (MUD) had a comparative 5-year OS with matched related donor (MRD) (78% vs. 64%, p = 0.21) (83). Siblings should also be genetically tested before donor selection due to the risk of carrying a similar mutation. Fludarabine-based reduced intensity conditioning (RIC) demonstrated less transplant-related mortality; however, it had a higher risk of graft failure, relapse, and mixed chimerism rates. Definitive treatment for syndromic HLH (CHS, GS, and HPS) also includes HSCT.

More recently, a deeper understanding of the molecular basis of HLH has led to the discovery of newer biological therapies for HLH. The central role of interferon-gamma (IFN-γ) in hyperinflammation in HLH led to the development of emapalumab, a monoclonal antibody against IFN-γ (84). It was FDA approved in 2018 for relapsed, refractory, or progressive familial HLH (85). Emapalumab is preferred over steroids, etoposide, and alemtuzumab in relapsed or refractory settings due to its better efficacy and safety profile. Emapalumab is given as an intravenous injection over 60 minutes every 3–4 days at a dose of 1–10 mg/kg, though it is started at a low dose of 1 mg/kg, increased as tolerated, and combined with dexamethasone for the entire duration of therapy. In a multicenter study, emapalumab and dexamethasone demonstrated a 63% overall response with eight days of median time to respond. Serum CXCL9 is a useful chemokine to assess the response produced by emapalumab (86). Alemtuzumab (median dose 1 mg/kg, range 0.1–8.9 mg/kg) divided over a median of 4 days (range 2-10) was used in a cohort of 22 pediatric and adult patients in relapsed/refractory settings; there was a partial response in 14 (86%) patients, with nearly three-fourths going for HSCT (87). Ruxolitinib, a Janus kinase (JAK) and Signal Transducers and Activators of Transcription (STATs) inhibitor, blocks downstream IFN- γ and other cytokine signaling without impairing T-cell cytotoxicity. Ruxolitinib administered as a salvage therapy for refractory HLH exhibited clinical improvement within 24 hours, a return of inflammatory markers to baseline within a month, and a successful HSCT (88). Wang et al. administered ruxolitinib with or without steroids to a cohort of 25 refractory/relapsed secondary HLH patients in children and adults. Overall response rate was 74% (14.7% complete, 58.8% partial) and survival rate was 56%, with a median response time of 22 weeks (89).Hansen et al. demonstrated that ruxolitinib can be safe and effective in elderly patients who are intolerant to etoposide (90). Immune checkpoint inhibitors like nivolumab has been demonstrated to restore T-cell immunity to EBV-HLH (91). Five of seven patients with relapsed/refractory EBV-HLH responded completely to nivolumab monotherapy in an adult cohort, with a median follow-up of 16 months (92).

Gene therapy trials for FHL are an active area of research. Pre-clinical studies have shown promising results for perforin deficiency and the UNC-13D type of FHLH (93–95). Takushi et al. optimized a lentivirus vector to restore Munc13-4 expression and degranulation capacity in both transduced FHL3 patient T cells and hematopoietic stem cells from the FHL3 (Jinx) disease model for FHL Type 3 (96). HSC gene transfer corrected the disease’s immunologic manifestations in the murine model in XLP (93).

COPA syndrome, being an autoinflammatory disorder, is treated with immunosuppressive medications. The main aim of treatment is to reduce the risk of pulmonary hemorrhage and related complications. Numerous immunosuppressive drugs are used, and most patients are often treated with a combination of medications. During exacerbations, corticosteroids, cyclophosphamide, and rituximab are utilized, while IVIG, methotrexate, azathioprine, hydroxychloroquine, and biologics such as etanercept have been attempted as maintenance therapy for interferonopathies. The recent association between COPA syndrome and IFN pathway activation led to the use of JAK inhibition to block interferon receptor downstream signaling in several patients. Baricitinib and ruxolitinib selectively inhibit JAK1/JAK2, whereas; tofacitinib inhibits JAK1/JAK3. Krutzke et al. described the successful use of baricitinib in a teenager with refractory COPA syndrome (97).Few COPA syndrome patients with severe pulmonary fibrosis and end-stage renal failure have been reported to have undergone lung and kidney transplantation (98, 99). Recent success with anti-interferon monoclonal antibodies (sifalimumab) in systemic lupus erythematosus suggests the effectiveness of this strategy (100). STING palmitoylation is essential at Golgi for STING activation, and inhibition of STING abolishes the type 1 interferon response. Therefore, these therapies may provide a new therapeutic option for COPA syndrome in the future (97, 101).

Currently, there is no cure for Vici syndrome or Takenouchi-Kosaki syndrome, and the treatment is mainly supportive care aimed at reducing the symptoms of the condition’s significant multi-system manifestation. Although autophagy inhibitors are used in cancer treatments, they are not effective in Vici syndrome. One toddler has been reported to have successfully undergone a liver transplant for immunodeficiency type 47 (65).

The definitive treatment for classic WAS is HSCT using a myeloablative conditioning regimen. Although milder disease variants like XLT can be monitored without a transplant in the absence of refractory thrombocytopenia/severe or recurrent infections, some experts recommend HSCT if a matched sibling donor is available. Allogenic transplant (MRD/MUD) outcomes are excellent for WAS, with a reported 5-year OS of 92% (102–104).

In conclusion, the number of intracellular trafficking disorders continues to increase as new diseases, and multi-system involvements are identified. Due to the rarity of the diseases and their overlapping symptoms, diagnosing these conditions could pose significant difficulties. Genetic tests are required for an accurate diagnosis. Currently, definitive treatment and a cure are only available for a few lymphocyte trafficking disorders, such as HLH and WAS. With a greater understanding of the underlying pathophysiologic mechanisms, safer and more effective targeted therapies or gene therapies can be designed to improve patient outcomes by addressing specific trafficking abnormalities.

## Author contributions

SHV: Drafted the article. JD: Critical review of the article. Both authors contributed to the article and approved the submitted version.
